# Isolation and characterization of cellulose nanocrystals from jackfruit peel

**DOI:** 10.1038/s41598-019-53412-x

**Published:** 2019-11-13

**Authors:** C. Trilokesh, Kiran Babu Uppuluri

**Affiliations:** Bioprospecting Laboratory, Centre for Bioenergy, School of Chemical and Biotechnology, SASTRA Deemed to be University, Thanjavur, 613 401 India

**Keywords:** Carbon cycle, Environmental impact, Crop waste, Nanoparticles

## Abstract

In the present work, sustainable nanomaterials, cellulose, and spherical cellulose nanocrystals (SCNCs) were isolated from the non-edible parts of jackfruit (*Artocarpus heterophyllus)*. Of the three different methods tested, sodium chlorite treatment produced the highest yield of cellulose, 20.08 ± 0.05% w/w (dry weight). Peaks observed in CP/MAS ^13^C NMR spectrum and FTIR frequencies revealed the presence of α-cellulose and absence of other biomass fractions like hemicellulose and lignin. XRD analysis showed a high crystallinity of 83.42%. An appearance of a sharp endothermal peak at 323 °C in DSC and decomposition patterns between 310–420 °C of TGA confirms the presence of cellulose. Further, Sulphuric acid hydrolysis was employed to produce SCNCs and examined by TEM for the morphology and by HPLC for the presence of glucose.

## Introduction

Cellulose is the amplest biopolymer on the earth^1,2^ and one of the extensively used polymers in food, pharmaceutical, and biofuel sectors^3,4^. Biomass-derived cellulose exhibits amorphous and crystalline forms with various regions like macro fibrils, fibers, pores, and micro & nanocrystals. At the molecular level, cellulose contains glucan chains and hydrogen bonds^5^.

Acid hydrolyzed cellulose generally produces CNCs of 20 × 100–200 nm size^6^. In addition to the commercial applications of cellulose, CNCs have gained tremendous attention in recent years for use in the environmental remediation technologies and pharmaceutical formulations. Ultralow density, tunable porous architecture, and outstanding mechanical properties of CNCs make them ideal for several applications. Nanocellulose has been used in the preparation of optical functional materials like chiral nematic, iridescent films, greenhouse plastics, anti-counterfeit technologies, and particle tracking^7^. CNCs based ultralight, durable and, flexible foams /aerogels for various applications are also being explored^8^. Nanocellulose has been widely used as a natural filler on multiple composites as it has high mechanical strength, modulus, 138–150 GPa; and tensile strength, 10 GPa^9^. Esterification of nanocellulose was reported for rendering hydrophobicity to aid easy dissolution in nonpolar solvents and polymer matrices^10^.

Sources like rubberwood, banana rachis, corn husk, macrophyte, rice husk, banana peels, jute, tree pruning, brown seaweed, and so forth were previously exploited for the isolation of cellulose and nanocellulose^11,12^. The present work focuses on the isolation of cellulose and cellulose nanocrystals (CNCs) from the non-edible parts of jackfruit.

The jackfruit tree is popular in India for its delicious seasonal fruit. It is widely cultivated in Kerala, Tamil Nadu, Karnataka, Andhra Pradesh, Maharashtra, Assam, Bihar, Orissa, and West Bengal regions with a total area of 13,460 ha^13^. A single jackfruit tree produces around 200–500 fruits annually with each fruit weighing around 23–40 kg^13^. The non-edible jack fruit peel generated by a single tree is around 2714–11800 kg per annum^13^. It is neither fit for human consumption nor animal feed and primarily comprises of cellulose, hemicellulose, and lignin. Though few studies have highlighted the use of jackfruit peel in environmental applications^14^, it is commercially not exploited. So a large volume has been disposed at landfills which cause environmental problems. The use of such renewable, sustainable, and cheap biomass for producing valuable second-generation products is the need of the hour in the perspective of economic and environmental significance. Hence the present work reports the isolation and characterization of a second-generation biomaterial, cellulose, and SCNCs from the jackfruit peel.

## Results and Discussion

### Compositional analysis

The jackfruit peel dried powder was examined for proximate, ultimate, and biochemical composition (Table 1). All the parameters were in good agreement with the reported literature. The composition and functional properties of holocellulose, extractives, and lignin may differ based on the structure and species of biomass. Moreover, only marginal differences were observed based on the seasonal and geographical variation^15,16^. The significant amount of holocellulose, 44.13% w/w (on d.w.b) and the entire carbon content encourage high-value applications of jackfruit peel. Minor proportions of lignin make it suitable for the fermentative production of biofuels. However, the structural and functional properties of high proportions of extractives require further investigations.

**Table 1:** Compositional analysis of jackfruit peel.

Sr. No	Component	Results of the present study	Reported in the literature	Reference
**Proximate analysis (%)**				
1	Total solids	91.66 ± 0.33	87.02 ± 0.42	Sundarraj and Ranganathan 2017
2	Moisture content	8.33 ± 0.33	12.98 ± 0.42	
3	Ash	7.67 ± 0.02	7.01 ± 0.19	
**Elemental analysis** (**%**)				
1	Carbon	42.20	63.6	Soetardji *et al*. 2014
2	Nitrogen	1.54	0.61	
3	Hydrogen	6.74	7.84	
**Biochemical Analysis** (**%**)				
1	Cellulose	20.08 ± 0.05	53.6	Selvaraju and Bakar 2017
2	Hemicellulose	24.04 ± 0.23.	22.5	
3	Lignin	1.85 ± 0.01	2.6	
4	Extractives	52.18 ± 0.03	15.3	

### Isolation of cellulose

**Figure 1:**
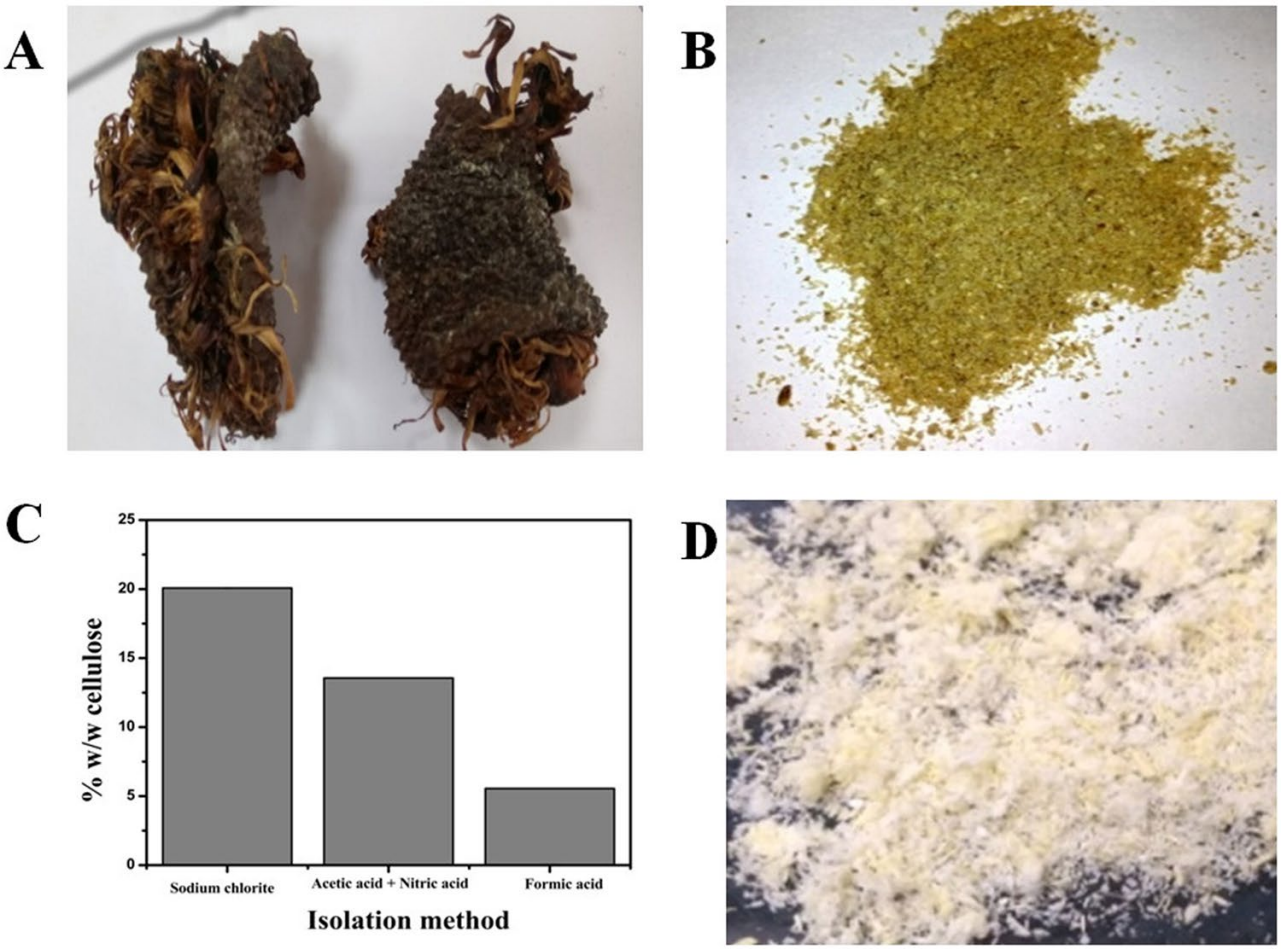
Images of (**A**) dried jack fruit peel (**B**) powdered jackfruit peel (**C**) cellulose yield from three different methods of isolation (**D**) bleached cellulose fibers using sodium chlorite treatment.

**Figure 2:**
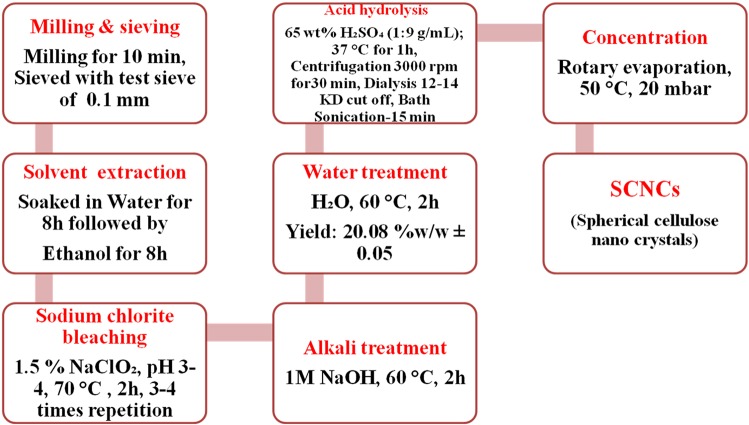
Scheme for the separation of cellulose and isolation of spherical cellulose nanocrystals from jack fruit peels.

Bleaching and pulping are two essential steps for the effective isolation of cellulose from the lignocellulosic biomass^17^. Out of the three different methods tested for bleaching, sodium chlorite yielded the maximum holocellulose (hemicellulose + cellulose), 44.13 ± 0.22% w/w. Hence the sodium chlorite method was used for the isolation of cellulose for all the analysis and characterization. Sodium chlorite is an excellent bleaching agent which aids in the removal of lignin and produces holocellulose^18–20^. The other two bleaching methods tested had chlorine-free eco-friendly chemicals such as hydrogen peroxide, acetic acid, nitric acid, and formic acid. Chlorine-free methods have been tried previously for the isolation of cellulose from oil palm empty fruit bunches, maize straw, rice husk, and wheat straw^21–24^. After bleaching, alkali treatment using sodium hydroxide at mild conditions helps to remove hemicellulose and isolate cellulose^17^. Initially, the untreated jackfruit peel powder was brownish-white in appearance (Fig. 1A,B). The presence of lignin, hemicellulose, extractives such as wax, cutin, and pectin, would render such appearance. Figure 1C depicts the final amount of cellulose from the tested methods. The combination of ethanol, water extraction, acid bleaching, and alkaline treatment ensured the removal of extractives, hemicelluloses, lignin, and produced cellulose (Fig. 1D). Figure 2 represents a detailed scheme for the isolation of cellulose and SCNCs by sodium chlorite treatment followed by sulphuric acid hydrolysis. In the current study, 20.08% ± 0.05 cellulose, 24.04% ± 0.23 hemicellulose, 1.85% ± 0.01 lignin, 52.18% ± 0.03 extractives, and 1.85% processing waste were determined in the jack fruit peel.

### SEM, TEM and zeta potential analysis

**Figure 3:**
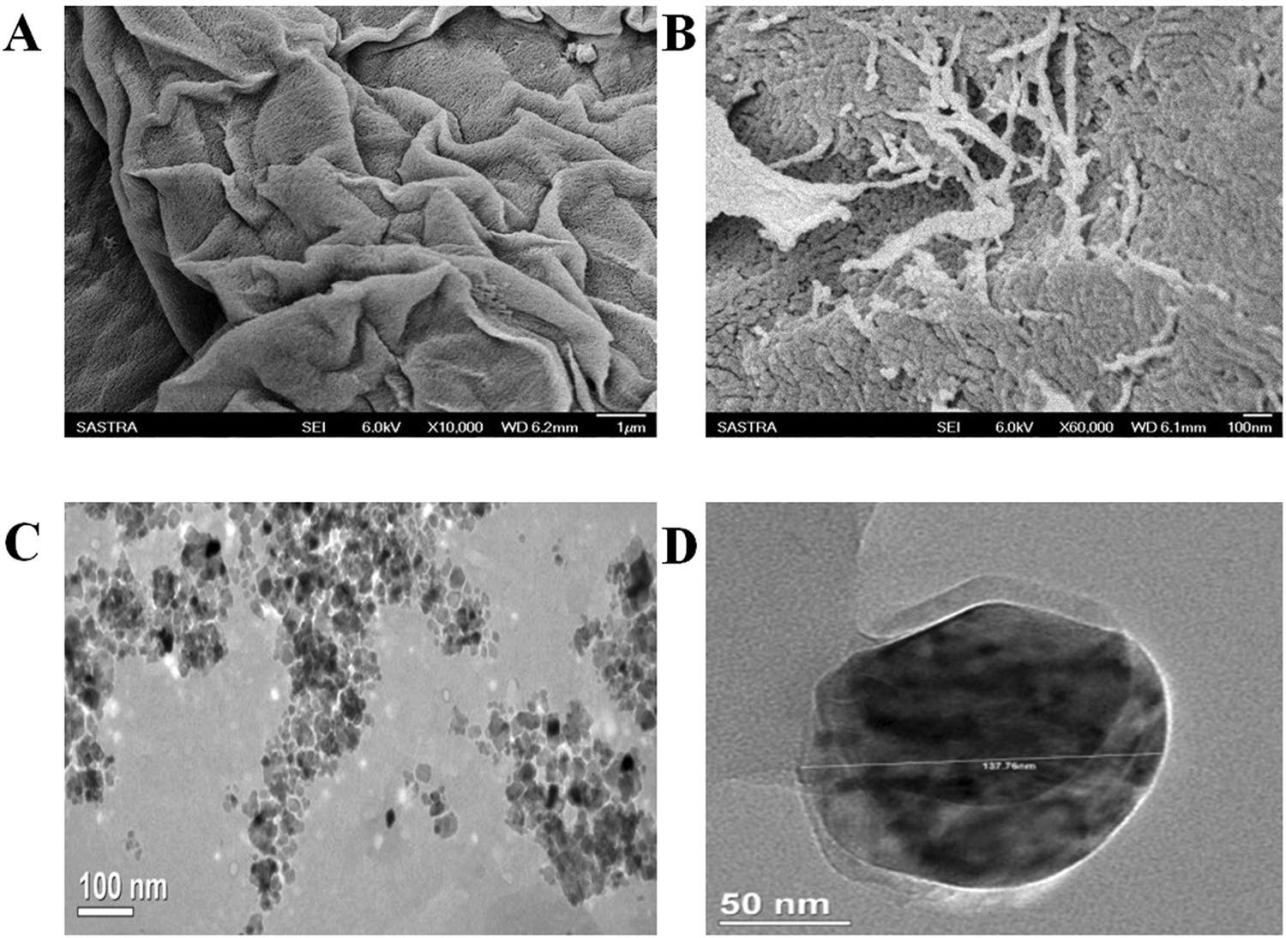
SEM micrographs of (**A**) cellulose fibers from sodium chlorite treatment (**B**) cellulose fibers with the width of 20–50 nm, TEM micrographs of (**C**) SCNCs (**D**) A single cellulose nanocrystal.

The functional properties of cellulose and SCNCs were influenced by their morphological features which depend on the source and method of hydrolysis. Morphological examination by SEM analysis revealed that the jackfruit peel contains curled and soft-flat shaped cellulose with rough pits (Fig. 3A). The curled shape provides a high surface area which favors the preparation of various composites and also for hydrolysis. Figure 3B shows the self-assembled structures of cellulose fibers due to the strong interfibrillar attraction between the surface hydroxyl groups.

Nanocellulose was obtained by removing the amorphous cellulose and fragmentation of cellulose fibers by sulphuric acid hydrolysis (Fig. 3C). Acid hydrolysis also produces spherical cellulose nanocrystals (SCNCs) which are novel and unique^25^. Hydrolysis of the inner amorphous cellulose fibers leads to the formation of spherical nanocellulose. In the present work the combined use of a strong bleaching agent, acid and ultrasonication would have led to the hydrolysis of inner amorphous regions of cellulose fibers. Figure 3D shows a soft, non-fibrous structured, non-agglomerated spherical shaped cellulose nanocrystals (SCNCs) with 130 nm diameter indicating the surface engraving and degradation nature of the hydrolysis^26,27^. In the present study, 7% w/w SCNCs was obtained from the isolated cellulose of jackfruit peel. The produced SCNCs has an average zeta potential −11.6 mv and size of 346 nm, which is in good agreement with the literature^28,29^. Nanocellulose has been reported for its large size variation^26,27^. Individual crystals would tend to aggregate due to water evaporation leading to larger particle size. The presence of aggregated and individual SCNCs in the acid hydrolyzed cellulose suspension led the particle size discrepancy as determined by TEM and zeta size.

### NMR analysis

**Figure 4:**
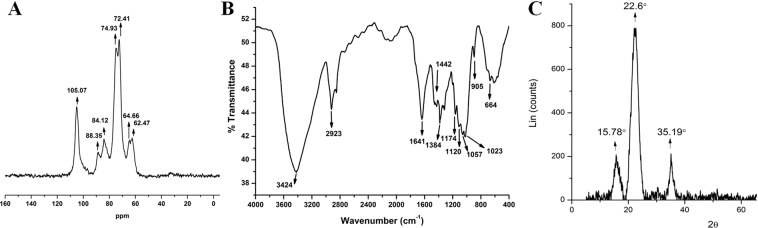
Characterization of the isolated cellulose from jackfruit peel (**A**) CP/MAS ^13^C NMR spectrum (**B**) FTIR spectrum (**C**) XRD analysis.

The solid-state NMR spectrum of the isolated cellulose was consistent with the literature^30^. The solid-state CP/MAS ^13^C NMR spectrum of cellulose (Fig. 4A) recorded peaks of α-cellulose at δ62.47 and 64.66 for C6, 72.41 and 74.93 for C2, C3 and C5, 84.12 and 88.35 for C4 and 105.07 for C1.

### FTIR analysis

The FTIR spectrum of the isolated cellulose displayed characteristic absorption patterns corresponding to the specific functional groups of α-cellulose (Fig. 4B) and was also in good agreement with the reported cellulose (Table 2). The peak at 1641 cm^−1^ may be formed due to the bending mode of adsorbed water^22,31,32^. The peak at 1442 cm^−1^ may be due to CH_2_ bending vibration^33^. The sharp transmittance peak around 1384 cm^−1^ represents a bending of OH groups^21^. The peak at 1174 and 1120 cm^−1^ corresponds to C-O asymmetric bridge stretching^21^. The peak at 1057 cm^−1^ may be due to C-O-C pyranose ring skeletal vibration^21^. The peak at 905 cm^−1^ is because of glycosidic linkages between sugar units^18^. The absence of shoulder peak at 1726 cm^−1^ indicates that there is no acetyl and uronic ester groups of the hemicelluloses or the ester linkage of lignin^30^.

**Table 2:** The isolated cellulose characteristic FTIR spectral assignments.

Sr.No	Assignment	Wavenumber (cm^−1^)		
		Isolated cellulose	Reported cellulose	References
1.	–OH groups stretching vibration	3424	3347–3450	Morán *et al*. 2008
2.	C–H stretching vibration	2923	2897–2900	Rosa *et al*. 2012
3.	H_2_O absorbed	1641	1632–1645	Szcześniak *et al*. 2008
4.	CH_2_ bending vibration	1442	1425–1468	He *et al*. 2018
5.	C–O–C glycosidic band stretching vibration	1057	1162–1172	Sun *et al*. 2004
6.	C–H rock vibration	905	896–905	de Oliveira *et al*. 2017

Similarly, the absence of peaks at 1413 and 1533 cm^−1^ indicates the absence of C=C of the aromatic ring of lignin^30^. So, the absence of peaks at 1413, 1533, 1726, 1244 cm^−1^ indicates the absence of hemicellulose and lignin. Thus, the FTIR spectrum confirms the purity of cellulose and the absence of hemicellulose and lignin.

### Crystallinity analysis by XRD

The mechanical and thermal properties of cellulose were dependent on the crystalline characteristics. Especially, the reinforcing capability and mechanical strength of cellulose are deciding factors for the use in environmental remediation technologies. The cellulose XRD diffraction patterns were recorded at 2θ = 15.7°, 22.6°, and 35.19° which are characteristic peaks for the cellulose corresponding to the lattice planes 110, 200 and 004 (Fig. 4C)^34^. The major crystalline peak was observed at 22.6° with an intensity of 100%, confirms the presence of crystalline cellulose^22,31^. From Eq. (1), 83.42% crystallinity index (CI) was calculated for the isolated SCNCs. Similar CI was reported for celluloses of poplar wood chips (50%)^35^, *Eucalyptus globulus* (55.3)^36^ and *E. Benthamii* (54%)^36^. Crystallite size is also supportive of the description of crystallinity of the cellulose. From Eqs (2, 3), the crystal size of the isolated cellulose was found to be 2.80 nm, and the interplanar distance was 0.21 nm. Similar results were found with the cellulose from *S. japonica*^33^.

### Thermal analysis

**Figure 5:**
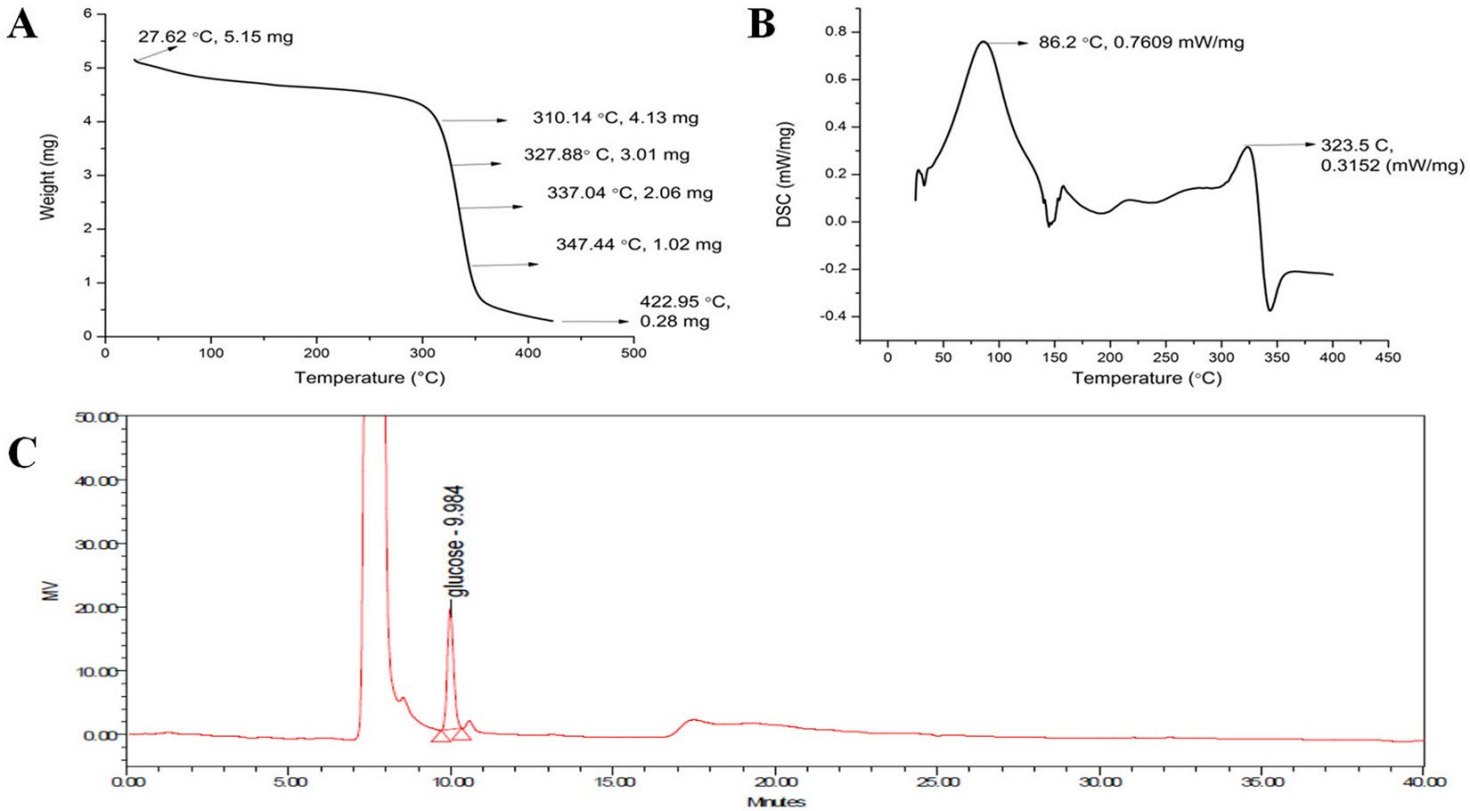
(**A**) TGA curve for cellulose (**B**) DSC curve for cellulose (**C**) HPLC chromatogram for hydrolyzed cellulose.

Thermal stability of the isolated cellulose was determined using TGA (Fig. 5A). Three stages of thermal degradation in the form of weight loss were observed. An initial steady-state decrease in weight till 150 °C is due to the evaporation of water. A sudden weight loss in the second stage during 200–380 °C is due to cellulose depolymerization. The final stage of rapid depolymerization of carbon residues occurred after 380 °C. An initial weight loss of 7% was observed below 100 °C, and after that, no decomposition was recorded till 180 °C. Thus TGA confirms the thermal stability of isolated cellulose and the absence of hemicellulose and extractives. Approximately 85% of the weight was lost by drying at 300–350 °C. A linear region of weight loss arose from 312–350 °C, which is the major characteristic thermal property of cellulose^37^.

DSC was performed to determine the energy consumption property of the isolated cellulose (Fig. 5B). A characteristic sharp endothermal peak at 323.5 °C was recorded^38^. No peaks corresponding to the hemicellulose (150 °C) and lignin (380–500 °C) were noticed. Thus, TGA and DSC results confirm the purity and excellent thermal stability of the isolated cellulose from the jackfruit peel.

### HPLC analysis

HPLC analysis of the SCNCs showed a single sharp peak corresponding to the standard glucose at 9.98 min (Fig. 5C). Approximately 67.5% w/w glucose was recovered from the SCNCs, and further optimization studies are under progress to enhance the hydrolysis and the yield of glucose.

## Experimental

### Materials

Jackfruit peel was collected from the same region of Thanjavur local market, India, throughout the entire work to avoid variations in nutritional content. Sodium Chlorite (NaClO_2_), Sodium hydroxide (NaOH), acetic acid (CH_3_COOH) was purchased from HIMEDIA Mumbai, India. Sulphuric acid (H_2_SO_4_) was obtained from SIGMA-ALDRICH, India. All other chemicals used in the study were AR grade and procured from MERCK, India.

### Compositional analysis

Jackfruit peels were washed first with tap water followed by distilled water to remove the adhered surface dust particles (Fig. 1A), and later air-dried for 48 h and milled. The dried powder was sieved with sieve numbers 80 (0.1 mm) and 20 (0.8 mm). Only those particles that passed through sieve number 20 and retained at sieve number 80 was used throughout the work (Fig. 1B). The amount of moisture, total solids, ash, and lignin were determined as per the NREL procedures^39,40^. The carbon, hydrogen, and nitrogen were determined using Elementar Vario EL III equipment, Germany.

### Isolation of cellulose

Three methods, namely sodium chlorite method, acetic acid plus nitric acid treatment and formic acid treatment, were screened for the maximum removal of non-cellulosic fragments and isolation of cellulose from the jackfruit peel powder.

### Method 1: Sodium chlorite method

Non-cellulosic fractions were completely removed, and cellulose was isolated as described elsewhere^34^. The powdered jackfruit peel was dewaxed with water followed by ethanol extraction in a Soxhlet apparatus for 8 h. The dewaxed powder was bleached using 1.5% w/v sodium chlorite in water (1:25 g/mL), pH 3.5 at 70 °C for 2 h. The bleaching process was repeated for four times till a white-colored holocellulose was formed and later subjected for alkali treatment for complete removal of the hemicellulose. Then the cellulose was suspended in water at 60 °C for 2 h to remove the excess sodium hydroxide. The pure cellulose obtained was filtered and dried.

### Method 2: Acetic acid plus nitric acid treatment

A slightly modified method suggested by Rehman *et al*., 2014 was followed for the isolation of cellulose^23^. The dewaxed biomass was treated with 5% w/v NaOH in water (1:30 g/mL) for 2 h to remove the hemicellulose. The treated biomass was bleached with a 10% v/v hydrogen peroxide at pH 11.8 for 12 h at 50 °C for 1:25 (g/mL) ratio. The bleached cellulose was purified further using 80% acetic acid (1:30 g/mL) and 65% nitric acid (1:4 g/mL). The purified cellulose was washed thrice with water and 95% ethanol and dried overnight at 50 °C.

### Method 3: Formic acid treatment

The hemicellulose in the biomass was removed using 3% v/v (1:25 g/ml) hydrogen peroxide and 10% w/v NaOH in water (1:25 g/mL) for 2 h at 65 °C. Later, 20% formic acid (1:10 g/mL) and 10% hydrogen peroxide (1:10 g/mL) in a 1:1 ratio was added and incubated at 85 °C for 2 h. The bleaching process using formic acid was repeated for 4–5 times until a white-colored product was obtained^23^.

### Isolation of nano-cellulose

The isolated cellulose was hydrolyzed using 65% H_2_SO_4_ at 1:9 (g/mL) ratio, 37 °C for 1 h^23^. The hydrolysis was stopped by the addition of double the amount of cold water. The cellulose suspension was later centrifuged at 3000 rpm for 30 min. The suspension was subsequently dialyzed using a dialysis membrane with cut off 12000 Da to 14000 Da to remove the excess sulphuric acid. Dialysis was continued till pH 5.5 was reached. The dialyzed suspension was ultrasonicated (Labman) for 15 min in bath sonication to get a uniform suspension of nanocrystals. The aqueous suspension was subsequently concentrated using rotary evaporator and dried (Buchi Rotavapor R-210, Switzerland).

## Characterization

### SEM analysis

SEM imaging of the isolated cellulose from jackfruit peel was studied using a JEOL JSM 6701 F microscope with an accelerating voltage of 6 kV. The sample was attached to an aluminum stub and was gold-sputtered to avoid electron charging effects.

### TEM analysis

The hydrolyzed cellulose suspension after dialysis was sonicated and analyzed for TEM. A drop of SCNCs solution was deposited on a copper grid, dried and imaged under a JSM 2100 F JEOL (Japan) microscope with an accelerating voltage of 200 kV. The images were processed using digital micrograph software.

### Zeta potential

Zeta potential and particle size analysis were measured using Malvern instruments, UK. Following parameters were followed for zeta potential measurement; temperature: 25 °C, count rate: 75.9 kcps and zeta runs: 100. For particle size analysis the temperature was kept at 25 °C, count rate of 125.9 kcps and duration of 80 s.

### NMR analysis

CP/MAS ^13^C spectrum of cellulose in D_2_O at room temperature (Bruker Avance HD 500 MHz spectrometer) was recorded at SAIF, IIT Madras, Chennai, India. The ^13^C NMR was operated at 125.76 MHz. The Proton 90° pulse was 4.30 µs; contact time was 3300 µs, recycle delay was 5 s and acquisition time was 0.02 s. Typically, 1024 scans per spectrum were recorded.

### FTIR analysis

A sample mixture of isolated cellulose, 2 mg, and dry KBr, 200 mg was prepared, pressed into a 16-mm diameter mold and FTIR spectra were measured on a Spectrum one, (Perkin Elmer, USA) instrument in the diffuse reflectance mode.

### XRD analysis

XRD pattern was recorded using Bruker D8 advance, equipment. The isolated cellulose was scanned in the range of 2θ = 5° to 90° at a step time of 0.2 s/step at 25 °C with Cu kα radiation, λ = 0.1540 nm. The Crystallinity index (CI) of dried cellulose was determined using the following equation^41^.

1 $${\rm{CI}}( \% )=100\ast \frac{{{\rm{A}}}_{{\rm{crystalline}}}}{{{\rm{A}}}_{{\rm{amorphous}}}+{{\rm{A}}}_{{\rm{crystalline}}}}$$

where A_amorphous_ is the area under the amorphous curve, and A_crystalline_ is the area under the sample curve.

The cellulose size was determined using Scherrer’s equation^34^

2 $${\rm{Crystal}}\,{\rm{size}}\,{\rm{L}}=k{\rm{\lambda }}/{\rm{\beta }}\,\cos \,{\rm{\theta }}$$

where λ = 0.1540 nm, k is the correction factor of 0.91, θ = diffraction angle in radians and β = full width at half maximum.

The d spacing of the cellulose can be determined using Bragg’s law^33^,

3 $$d=n\lambda /(2\,\sin \,\theta )$$

where d is the interplanar distance between lattice planes, θ is the scattering angle in degrees, n is a positive integer and λ is the wavelength of the x-ray

### TGA analysis

To determine the thermal stability and decomposition pattern of isolated cellulose, Thermogravimetric analysis (TGA), was performed in an SDT Q600 instrument. For each measurement, approximately 5 mg of the sample was used. Patterns were recorded under a nitrogen atmosphere at a flow rate of 100 mL/min by heating the material from room temperature to 500 °C at a heating rate of 20 °C/min.

### DSC analysis

DSC was performed using a NETZSCH DSC 214 Polyma instrument. Approximately 5.3 mg of cellulose was taken in a concavus pan crucible with pierced lid. The sample was heated up to 400 °C at a heating rate of 25 °C/min under a nitrogen environment at a flow rate of 40 mL/min.

### HPLC analysis

HPLC analysis was conducted to determine the presence of glucose and the absence of hemicellulose sugars in the SCNCs. Briefly, the depolymerized cellulose was dissolved in the mobile phase (80% acetonitrile and 20% water) and loaded into a spherisord amino column (4.6 × 250 mm) of Waters HPLC system. The system was equipped with a Waters 2535 quaternary gradient pump and Waters 2414 Refractive index detector. Isocratic elution was performed after injecting 10 µL of the sample at a mobile phase flow rate of 1 mL/min, a column temperature of 35 °C, and a detector temperature of 30 °C.

## Conclusion

In the present work, cellulose and SCNCs were successfully isolated from the non-edible jackfruit peel using sodium chlorite treatment followed by sulphuric acid hydrolysis. Approximately 44% w/w (on a dry weight basis) of holocellulose was isolated, of which 20% w/w was cellulose. The isolated cellulose was characterized for its morphology, functional, crystal, thermal properties by SEM, FTIR, NMR, XRD, DSC and TGA. The SCNCs were characterized for its size, surface charge and monomer analysis by TEM, Zeta potential, zeta size and HPLC. The isolated cellulose and SCNCs exhibited similar characteristics reported in the literature and was found to be devoid of hemicellulose and lignin. The present results indicated the significance of the valorization of jackfruit peel. Several potential applications of CNCs in the field of food, paper, paints, optics, pharmaceutics, environment remediation, composite synthesis, and so forth have been reported. Hence the isolated cellulose and SCNCs from jackfruit peel waste can be employed for such applications.

## Data Availability

All data generated or analyzed during this study are included in this published article.
